# Foreign Summary

**Published:** 1839-08-10

**Authors:** 


### FOREIGN SUMMARY.

*Clinical Observations on Fever.* By Charles Lendrick, M. D. T. C. D., Queen's Professor of the Practice of Medicine in the School of Physic in Ireland.—Fever was described in the synopsis of my course of lectures on the *Practice of Medicine*, published in the year 1833, as a disease dependent on "*morbid actions* NOT *referrible to any particular structure or system.*" In thus denying the essential connexion of fever with organic lesions, or morbid changes of the fluids, I did not, nor do I now, claim originality. In my opinion, the priority as to time, in alleging a fact, is but a poor test of professional knowledge; and the palm ought to be assigned, not to him who (perhaps accidently) stumbled on the truth in the first instance, but to those who, in opposition to fashionable theories and received doctrines, steadily upheld what proved to be established on the most solid grounds. In making this remark, I wish to be understood as referring to the expressed opinions of able and experienced practitioners in Dublin, who, for probably half a century, have, through evil report and good report, denied the hypotheses of both solidists and fluidists, as explaining the source of *fever*.

I am not an advocate for *authority* in medicine; yet I confess that when, many years ago, I considered the extensive opportunities that Dublin affords to the practitioner, in fever—the enormous prevalence of that disease in our metropolis—the excellence of its principal institution (in Cork street) for the reception of fever patients—and the consummate skill and experience of such men as Quin, Purcell, Harvey, Plunkett, Perceval, &c., I felt, even at a very early period of my career as a medical man, an inclination to doubt the theories so imposingly put forward by authors whose opportunities were so much inferior to those of the distinguished practitioners I have just mentioned, and which, in my opinion, were the less deserving of confidence, from the overweening confidence with which they are urged.

On perusing, several years since, Travers' work on Constitutional Irritation, I was struck by the coincidence between his statements and my own experience. I asked myself the question repeatedly—"Did I not know all this before?" It seemed to me that I had learned nothing new, as is the case with most persons *after* they have become acquainted with the discoveries of others. Medical discovery, however, generally merely renders clear what was formerly obscure; and we are too apt to confound the twilight of our own recollection with the clear elucidation of the subject by others.

When I had methodized my thoughts, after a perusal of Travers' work, and its comparison with (or rather confirmation by) what I had observed, it seemed to me that there was no important distinction (contagion excepted) between fever and what he termed *constitutional irritation.* A fracture—a contusion—a wound—any injury, however slight, appeared to be capable of producing all the phenomena of *fever.* Delirium—coma—convulsions—a surface, hot, cold, or flooded by perspiration—excessive and depraved secretions, or a stoppage of these secretions—every imaginable variation in the state of the human economy was capable of being produced by the most trifling irritation of the *nervous system*, under peculiar circumstances.

Here is an important point established in our analysis. A similarity of effect goes a certain way in proving a similarity of cause. The identity, however, of fever with constitutional irritation admits of further proof.

1st. In both diseases the influence of an *external* cause is requisite to produce the effect. In that of constitutional irritation, the impression of a local irritant on the nervous system is obvious, from the effect of the external injury. In the case of fever, the nature of the influence of a cause external to the system is much more obscure; but the necessity of such a cause in order to produce the usual effect is undeniable. What are called the "accessory" causes of fever—cold, mental anxiety, intemperance, &c. are quite inadequate *in themselves* to its production; they may establish an inflammatory or nervous disease, but they will not cause *typhus fever*, unless typhus be prevalent in the district at the time, or unless the patient has been exposed to contagion. *Then*, the means which on other occasions produce other diseases will engender typhus fever of the worst description. In *contagion*, and what are called *epidemic* and *endemic* agency, whether combined or uncombined with the *accessory* causes of fever, we therefore, have a principle, which in this disease is equivalent to the influence of an external injury in producing constitutional irritation.

2dly. In both fever and constitutional irritation, we observe the same influence of the constitutional powers of the individual affected. While all the symptoms of this morbid state are produced by an external injury, a similar, or a much greater injury, will be borne by another patient with not the tenth part of the constitutional disturbance. So in the case of fever, several persons will be similarly situated as to the influence of pestilential agency or contagion; yet one will be attacked by typhus fever of intense severity, another will labour under merely a *febricula*, while a third will escape altogether.

3dly. Organic changes take place in both diseases under similar circumstances. If the patient be predisposed to any organic affection, it is sure to display itself during the perturbation of the system consequent on the establishment of fever or constitutional irritation. This will happen even at an early period, and to a considerable amount, if the patient has been much exposed to the operation of those agents, cold, vicissitudes, &c., which are accessory in the production of the febrile affection, and which exert the principal influence in forming organic disease.

Constitutional irritation thus renders us powerful pathological assistance, by showing the capability of an external and remote irritation of the nervous system to produce *organic disease*, as well as a constitutional disturbance similar to fever. A man receives a bruise on his head; severe constitutional disturbance supervenes; he displays symptoms of local disease in the thoracic or abdominal viscera; he dies, and the expected organic changes are discovered. Here no one can say that pneumonia or hepatitis was the *cause* of the disease, which is distinctly referrible to an obvious injury inflicted on a limb. Hence, we arrive at the conclusion that the *occasional* appearance of certain organic changes in *fever* ought not to induce us to refer the cause of this disease to them. We cannot trace out its exciting cause, contagion, &c., with such facility as in the case of external injury; but the circumstance that there *is* an external cause in operation, (although more securely,) and with nearly similar results, in the one case as in the other, and that in both there are the same variations as to the occurrence of organic affections, whether as to this taking place at all, or in any particular form, afford confirmation to the great similarity (or nearly identity) of fever and constitutional irritation. Now as the latter is clearly referrible to an external impression made on the nervous system, modified by the constitutional idiosyncrasy of the individual, and by the operation of what are termed accessory causes, ought not FEVER to be referred to a similar source—with the substitution of contagion, or epidemic, or endemic agency, for injury, &c.?

Some say, "Any pathology rather than none." This I deny. The person who knows by experience merely that a certain remedy applied in a certain way will cure a disease, and who proceeds on this principle, is a far safer practitioner than he who adopts erroneous opinions, and *acts* on them. The reason that false pathology has not done so much mischief as might have been expected is, that practitioners often theorise about disease in one way, and practise in another. Their theory is unfounded, but their practice, based on their experience, and not on their avowed principles, is correct.

Among the many physicians who have been distinguished in Dublin for their skill in the treatment of fever, none perhaps have attained to so great a celebrity as the late Dr. Harvey, nor has there appeared since any one so conspicuous for the successful management of what seemed to be desperate cases. I endeavoured, at an early period of my professional career, to make myself acquainted with his rules of practice; and the most accurate information I could acquire was, that his great secret consisted in doing "*very little.*" It was a sarcastic remark of his, that "the patient was in greater danger from the doctor than from the disease."

It is not unlikely that a modification of this remark of Dr. Harvey is applicable to many diseases besides fever. It is still in reserve for us to learn, how disease will subside without the daily administration of medicine, or how far it is a provision of nature that it shall run its course, and then terminate favourably. For instance, a principle most confidently inculcated formerly, that the tendency of cholera is always to a fatal termination, and that the patient is never saved but by art, was disproved by the fact frequently observed in Ireland, that paupers left in the last stage of cholera asphyxia to die in a ditch, recovered notwithstanding, and without the use of any medicine but cold water.

"What is to be *done*," is a frequent question at medical consultations. It is often, however, of more consequence to know what is *not* to be done; and the practitioner often cannot display greater skill in fever than by knowing when to do nothing. By adopting the negative mode of treatment in the great proportion of cases, and by interfering in the others, only to the amount actually indispensable, during my attendance at Sir Patrick Dunn's Hospital, when fever was unusually prevalent two years ago, I lost but two patients; one of whom was a confirmed drunkard, and the other laboured under long-established emphysema of the lungs, complicated with bronchitis. For the latter disease, rather than fever, she was admitted into the hospital.

It is fully established, that patients will recover in a great majority of instances, from typhus fever, by the almost unassisted powers of nature. In many such cases, especially among the lower orders of society, such is the torpor of the functions, partly from the disease, and partly from previous habits, that a medical attendant can scarcely do harm by any practice confined within reasonable bounds; and whether he bleeds and purges on the one hand, or uses bark and wine on the other, provided he does so moderately, he will not *prevent* the patient's recovery. This fact has not only led to the undeserved reputation of alleged specific modes of treatment in fever, by a coincidence of spontaneous recovery with their supposed effects, but has also caused an opprobrium to our profession, namely, that statistical returns prove that the average amount of mortality is nearly the same under one mode of treatment as another. The reason of this, however, is, that the *average* mortality is influenced by the *large* proportion of cases, which are of the description I have mentioned, where almost any treatment or no treatment is equally successful as to the result. It is in the *small* proportion that medical skill and the effects of medical treatment are shown; and these, from their fewness, but little affect statistical returns. In such cases the practitioner is constantly between Scylla and Charybdis; and whether the necessities of the case require bleeding, purging, mercury, wine, or other stimulants, whatever is done beyond what is actually *necessary*, is done injuriously.

Typhus fever presents itself in two forms; typhus, properly so called, and the synochus of Cullen, where local inflammation to a greater or lesser extent is combined with typhus. The distinction of such cases, and the mode of treatment, will be referred to hereafter; for the present, I cannot too strongly urge the danger of being led astray by that perturbation of the system which once led to the hypothesis of a fermentation of the fluids; and under the operation of which, local disease, which in itself might require active and special treatment, is liable to be simulated. Thus, with disturbance of local functions, or determination of blood to various parts, unusual secretions take place in the respiratory organs, as elsewhere; and which, on the application of auscultation, seem to depend on organic disease. I have been repeatedly assured by others, after the application of the stethescope, that the lungs were softening, or that latent pneumonia was present; and yet the physical signs subsided, without any direct treatment, on convalescence from fever being established.

I particularly distrust *crepitus*, when existing merely in the *gravitating* portion of the lung. I have repeatedly observed it at the back of the thorax, when I am convinced from the progress of the case, that it depended merely on a general secretion which had become accumulated there in consequence of the supine posture of the patient. I therefore rarely direct any special treatment for *mere* crepitus, when occurring in typhus fever; inasmuch as there is less danger in allowing pneumonia, if present, to further develope itself, than to venture on bleeding, mercurialization, or the action of the tartarized antimony, *unnecessarily*, in *typhus*.

[*To be continued.*]

